# Acute Failure of Catheter Ablation for Ventricular Tachycardia Due to Structural Heart Disease: Causes and Significance

**DOI:** 10.1161/JAHA.113.000072

**Published:** 2013-06-21

**Authors:** Michifumi Tokuda, Pipin Kojodjojo, Stanley Tung, Usha B. Tedrow, Eyal Nof, Keiichi Inada, Bruce A. Koplan, Gregory F. Michaud, Roy M. John, Laurence M. Epstein, William G. Stevenson

**Affiliations:** 1 Arrhythmia Unit, Cardiovascular Division, Brigham and Women's Hospital, Boston, MA (M.T., P.K., S.T., U.B.T., E.N., K.I., B.A.K., G.F.M., R.M.J., L.M.E., W.G.S.)

**Keywords:** catheter ablation, failure, outcome, structural heart disease, ventricular tachycardia

## Abstract

**Background:**

Acute end points of catheter ablation for ventricular tachycardia (VT) remain incompletely defined. The aim of this study is to identify causes for failure in patients with structural heart disease and to assess the relation of this acute outcome to longer‐term management and outcomes.

**Methods and Results:**

From 2002 to 2010, 518 consecutive patients (84% male, 62±14 years) with structural heart disease underwent a first ablation procedure for sustained VT at our institution. Acute ablation failure was defined as persistent inducibility of a clinical VT. Acute ablation failure was seen in 52 (10%) patients. Causes for failure were: intramural free wall VT in 13 (25%), deep septal VT in 9 (17%), decision not to ablate due to proximity to the bundle of His, left phrenic nerve, or a coronary artery in 3 (6%), and endocardial ablation failure with inability or decision not to attempt to access the epicardium in 27 (52%) patients. In multivariable analysis, ablation failure was an independent predictor of mortality (hazard ratio 2.010, 95% CI 1.147 to 3.239, *P*=0.004) and VT recurrence (hazard ratio 2.385, 95% CI 1.642 to 3.466, *P*<0.001).

**Conclusions:**

With endocardial or epicardial ablation, or both, acute ablation failure was seen in 10% of patients, largely due to anatomic factors. Persistence of a clinical VT is associated with recurrence and comparatively higher mortality.

## Introduction

Ventricular tachycardia (VT) is a marker for increased mortality and reduces quality of life in patients who have implanted defibrillators and structural heart disease.^1–2^ Catheter ablation is helpful in reducing recurrent VT in many patients, but the procedure fails acutely in 10% to 20% of patients, and overall approximately half of patients in multicenter trials will experience at least 1 VT recurrence after ablation.^3–6^ The presence of inducible monomorphic VT after ablation is associated with worse outcomes.^3,7^ The reason for acute ablation failure is sometimes difficult to assess and often not reported. Inability to identify the VT focus and achieve ablation, likely related to anatomic obstacles, is a potentially important cause. The frequent presence of multiple VTs and inability to clearly separate those that have previously occurred spontaneously, designated “clinical VTs,” from other inducible VTs complicate assessment of the acute effect of ablation. The aim of this study is to identify causes for acute ablation failure as indicated by persistent inducibility of a clinical or presumed clinical VT. We also related this short‐term result to outcome during follow‐up.

## Methods

### Patient Selection

From 2002 to 2010, 518 consecutive patients (84% male, 62±14 years) with structural heart disease underwent first ablation for sustained VT at our institution, and they constitute the study population. We classified underlying cardiomyopathy as ischemic cardiomyopathy, dilated cardiomyopathy, arrhythmogenic right ventricular (RV) cardiomyopathy (ARVC), hypertrophic cardiomyopathy, cardiac sarcoidosis, valvular heart disease, or congenital heart disease, as previously described.^8^ Each patient gave written informed consent. Studies and data collection were performed according to protocols approved by the Human Research Committee of Brigham and Women's Hospital.

### Electrophysiological Study

Ventricular mapping and radiofrequency ablation were performed with saline‐irrigated or nonirrigated 8‐mm‐tip catheters as previously described.^9^ Electroanatomic mapping was performed with the CARTO mapping system (Biosense Webster). Bipolar electrocardiograms (ECGs) were band pass filtered from 30 to 500 Hz and digitally recorded along with a 12‐lead surface ECG by using the Cardiolab EP system (General Electric Healthcare). Electrogram amplitudes of ≤0.5 mV were defined as dense scar and voltages of >0.5 mV and of ≤1.5 mV as scar borderzone.^10^ Programmed stimulation for initiation of VT used 1 to 3 extrastimuli scanned to refractoriness or a minimum coupling interval of 180 ms, applied after a basic drive of 600 ms and then 400 ms from 2 RV sites. Isoproterenol was used if VT was exercise related and usually in ARVC.

Ablation targeted all clinically relevant VTs, which were defined as sustained monomorphic VTs that had a longer or equivalent cycle length compared with documented spontaneous VT. VTs of shorter cycle lengths were targeted at the discretion of the investigator. Hemodynamically tolerated VTs were mapped and ablated during VT. Target sites for ablation were reentry circuit isthmus sites based on entrainment mapping (pacing entrained the VT with concealed fusion and a postpacing interval within 30 ms of the VT cycle length) or, if none were identified, sites with an isolated mid‐diastolic potential or presystolic ECGs.

Electroanatomic mapping for unstable, “unmappable” VTs was performed during sinus rhythm. Ablation targeted presumptive channels and exits within the low‐voltage area as identified from a paced QRS morphology similar to the VT QRS morphology, wide fractionated potentials, or isolated late potentials during sinus or paced rhythm where pacing captured, particularly if the S‐QRS interval was >40 ms, consistent with abnormal conduction. Furthermore when these sites were adjacent to a valve annulus or region of electrically unexcitable scar, ablation lesions were extended to the unexcitable area in the hope of dividing reentry circuit paths.^11^ When possible, we assessed ECGs and entrainment at the initial sites of interest with initiation of a short episode of VT. This was particularly attempted when initial substrate ablation failed to abolish inducible VT. If no low‐voltage area was identified, ablation was attempted at the likely exit region identified as sites with presystolic ECGs during VT or where pace‐mapping resembled the VT QRS. Applications were repeated at target areas until unipolar pacing at 10 mA at 2‐ms stimulus strength failed to capture.^12^ If endocardial ablation failed, percutaneous epicardial mapping and ablation were performed as previously described,^13^ provided that epicardial access could be achieved at the same or a subsequent procedure. Epicardial ablation via subxiphoid epicardial window or surgical ablation could be considered in patient who had a history of cardiac surgery rendering pericardial access more difficult.^14–15^

### Definitions and End Points

Clinical VTs are defined as having a rate within 20 ms and QRS morphology agreeing in bundle‐branch block configuration in V1, frontal plane axis, and transition with a spontaneously documented VT. Presumptive clinical VTs had a cycle length within 20 ms of a spontaneous VT, without available complete 12‐lead ECGs to compare spontaneous QRS morphology. VTs of shorter cycle lengths were targeted at the discretion of the investigator. After ablation, VT inducibility was assessed using programmed electrical stimulation with up to 3 extrastimuli from 2 RV sites with or without isoproterenol infusion. Ablation success was defined as the absence of any inducible monomorphic VT at the end of the ablation procedure or abolition of the clinical or presumed clinical VT, but other VTs remained inducible. Acute ablation failure was defined by inability to render a clinical or presumed clinical VT noninducible. Renal insufficiency was defined as an estimated glomerular filtration rate <60 mL/min per 1.73 m^2^.^16^

### Data Collection and Follow‐up

Data were collected from a centralized system containing records of all patients treated and followed at Brigham and Women's Hospital and all associated Partners Healthcare sites. These records include emergency department visits, outpatient clinic visits, data recorded during inpatient care, and follow‐up progress notes from referring physicians monitoring out‐of‐area patients. In addition, referring cardiologists and primary care physicians were contacted for clinical follow‐up of their patients if necessary. Mortality was determined by interrogation of the Social Security Death Index.

### Statistical Analysis

Continuous variables are expressed as mean±SD. Unpaired Student *t* test or Mann–Whitney *U* tests when distribution was not normal was used for continuous variables. Categorical and binary variables are presented as frequencies (percentages). Intergroup comparisons of categorical variables were conducted by means of χ^2^ analysis. Multivariate analyses used logistic regression analysis to assess predictors of ablation failure. A 0.10 level of significance was used for variable entry and removal from the stepwise models. The following variables were included as candidates for entry into the stepwise models: ischemic cardiomyopathy, VT storm: defined as ≥3 episodes of VT within 24 hours, history of prior VT ablation, number of VT morphologies induced, and New York Heart Association (NYHA) functional class III or IV. Survival curves were created and compared using the Kaplan–Meier method, and probability estimates were based on the log‐rank test. Cox proportional hazard models were used to assess predictors of outcomes. The following variables were included: age, left ventricular (LV) ejection fraction, ischemic cardiomyopathy, NYHA class III or IV, renal insufficiency, VT storm, number of failed antiarrhythmic drugs, history of prior VT ablation, and ablation failure. Significance was defined as *P*<0.05. All 518 patients were included in multivariable and survival analyses. In pairwise comparison among 3 groups, a *P* value of 0.05/3 (equivalent to a Bonferroni‐adjusted [3 tests] *P* value of 0.05) was considered significant. Statistical analyses were performed using SPSS, version 20.0 (IBM). The authors had full access to the data and take full responsibility for the integrity of the data.

## Results

### Baseline Characteristics

The acute procedure outcome was classified as a failure in 52 (10%) and a success in 383 (74%), and postablation attempts to reinduce VT were not performed to avoid aggravating hemodynamic status in 83 (16%) patients. The patients who did not have a postablation attempt to reinduce VT had more advanced disease (Table 1). Compared with the success group, the failure group had a greater number of prior failed ablation procedures, more patients with a history of VT storm, longer total fluoroscopic times, but shorter total radiofrequency ablation time (Table 2). Of 518 procedures, over time (before versus after January 2005), acute failure decreased from 14% to 9% (*P*=0.09), use of an irrigated catheter increased from 44% to 90% (*P*<0.001), and use of epicardial ablation increased from 7% to 15% (*P*=0.01). The incidence of acute ablation failure did not differ according to underlying heart disease (9% [28/314] in ischemic heart disease, 15% [17/114] in dilated cardiomyopathy, 7% [1/31] in ARVC, 18% [2/11] in sarcoidosis, 0% [0/8] in hypertrophic cardiomyopathy, 7% [1/14] in congenital heart disease, and 12% [3/26] in valvular heart disease; overall *P*=0.33). LV and RV substrate maps were performed in 440 (85%) and 264 (51%) patients, respectively. The proportion of patients with LV and RV scar who had persistent inducibility of the clinical VT were similar (11.4% versus 10.6%, *P*=0.98).

**Table 1.** Clinical Variables Between Patients With and Without Postablation Test

	Not Tested (n=83)	Tested (n=435)	*P* Value
Patient Characteristics			
Age, y	63±13	61±14	0.18
Male sex	69 (83)	364 (84)	0.90
LVEF, %	29±13	34±15	0.01
LVEF ≤40%	66 (80)	283 (65)	0.01
Ischemic heart disease	53 (64)	264 (61)	0.59
LVDd, mm	63±14	61±11	0.72
NYHA class III or IV	39 (47)	94 (22)	<0.001
History of cardiac surgery	23 (28)	180 (41)	0.02
No. of failed ablations	0.7±0.9	0.5±0.8	0.07
Renal insufficiency	42 (50)	144 (33)	0.004
VT storm	16 (19)	111 (26)	0.23
No. of failed AADs	2.5±1.2	2.2±1.2	0.03
ICD	79 (95)	376 (86)	0.03
Procedural details			
Cooled‐tip catheter	65 (78)	345 (79)	0.84
No. of VT morphologies induced	2.9±1.9	2.7±1.8	0.23
Only unmappable VT	37 (45)	131 (30)	0.01
Only mappable VT	10 (12)	112 (26)	0.007
Total fluoroscopic time, min	43±22	44±25	0.82
Total RFA time, min	33±22	31±22	0.38

Data are presented as mean±SD or n (%). LVEF indicates left ventricular ejection fraction; LVDd, left ventricular diastolic dimension; NYHA, New York Heart Association; VT, ventricular tachycardia; AAD, antiarrhythmic drug; ICD, implantable cardioverter‐defibrillator; RFA, radiofrequency ablation.

**Table 2.** Clinical Variables Between the Patients With Ablation Success and Failure

	Ablation Success (n=383)	Ablation Failure (n=52)	*P* Value
Patient characteristics			
Age, y	61±14	63±14	0.31
Male sex	322 (84)	42 (81)	0.55
LVEF, %	34±16	32±12	0.69
LVEF ≤40%	248 (65)	35 (67)	0.72
Ischemic heart disease	236 (62)	28 (52)	0.35
LVDd, mm	60±11	61±9	0.55
NYHA class III or IV	78 (20)	16 (31)	0.09
History of cardiac surgery	159 (42)	21 (40)	0.88
No. of prior failed ablations	0.5±0.8	0.7±0.9	0.04
Renal insufficiency	124 (32)	17 (33)	0.96
VT storm	92 (24)	19 (37)	0.04
No. of failed AADs	2.2±1.2	2.1±1.2	0.42
ICD	332 (87)	44 (85)	0.68
Procedural details			
Cooled‐tip catheter	306 (80)	39 (75)	0.41
No. of VT morphologies induced	2.6±1.5	3.2±2.9	0.39
No, of mappable VTs	1.0±1.1	1.2±1.4	0.29
Total fluoroscopic time, min	43±25	56±24	<0.001
Total RFA time, min	32±22	25±21	0.02

Data are presented as mean±SD or n (%). LVEF indicates left ventricular ejection fraction; LVDd, left ventricular diastolic dimension; NYHA, New York Heart Association; VT, ventricular tachycardia; AAD, antiarrhythmic drug; ICD, implantable cardioverter‐defibrillator; RFA, radiofrequency ablation.

### Failure According to Endocardial and Epicardial Ablation

**Figure 1.**
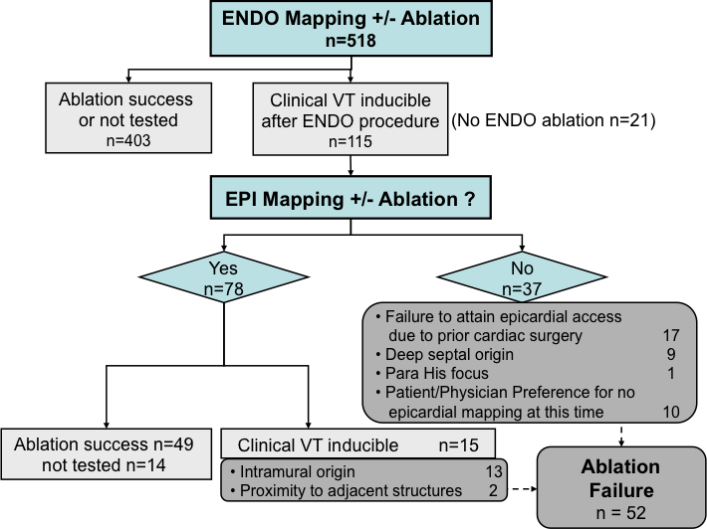
Flow chart and causes of ablation failure. After endocardial mapping, 101 (23%) had failed ablation. Epicardial mapping was performed in 64 patients, which resulted in acute procedural success in an additional 49 patients. Thus, 52 (12%) were acute failures. ENDO indicates endocardial; EPI, epicardial; VT, ventricular tachycardia.

**Figure 2.**
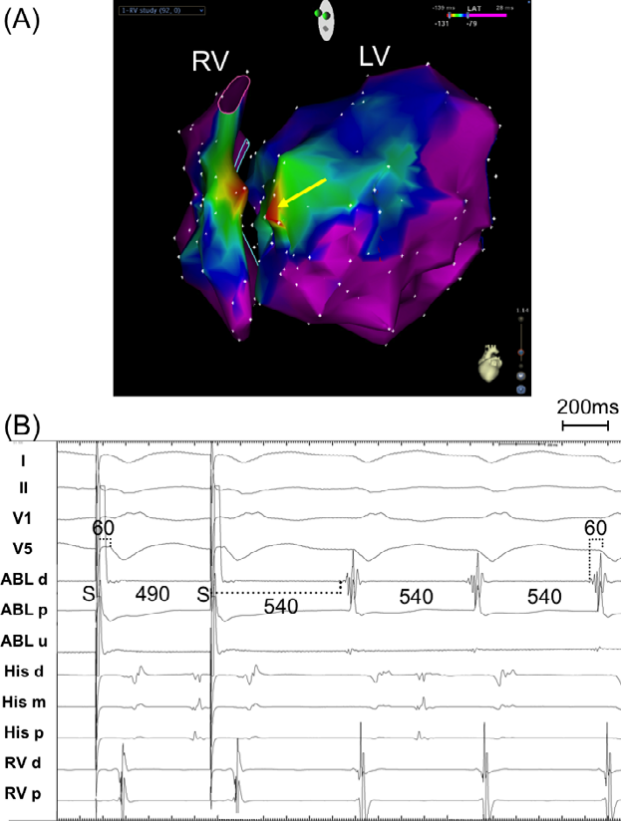
Example of septal origin. (A) Merged RV and LV activation maps of VT indicating the earliest activation during VT is located at the mid septum. (B) Sustained monomorphic VT with a cycle length of 540 ms is present. The first 2 beats of a train of stimuli at a cycle length of 490 ms is shown. The VT is continually reset without an alteration in the QRS morphology consistent with entrainment with concealed fusion. The stimulus‐to‐QRS interval is 60 ms. As shown in the last beat of the tracing, the electrocardiogram onset at the recording site occurs 60 ms before the QRS onset. Thus, the stimulus‐to‐QRS interval matches the electrocardiogram‐to‐QRS interval. The postpacing interval matches the VT cycle length of 540 ms. These findings are consistent with pacing at a reentry circuit site near an “exit”. ABLd indicates bipolar intracardiac recordings from the distal electrode pair of the mapping catheter at left ventricular site; ABLp, those from the proximal pair; RV, right ventricle; LV, left ventricle; VT, ventricular tachycardia; Uni, unipolar recordings; d, distal; m, middle; p, proximal.

A flow chart indicating causes of acute ablation failure is shown in Figure 1. Endocardial mapping with or without ablation was performed but failed to abolish clinical VTs in 115 (22%) patients; endocardial ablation lesions were applied but failed in 93; endocardial mapping was performed but no ablation lesions applied due to absence of a target site in 21 patients and a para‐His VT site in 1 patient. Epicardial mapping was performed in 78 patients. A successful epicardial ablation site was identified in 49 and an attempt to reinduce VT was not performed in 14, leaving 52 (10%) patients with acute failure defined as persistently inducible clinical or presumptively clinical VT. In 13 patients, both endocardial and epicardial mapping failed to identify a target site, suggesting that the circuit was intramural. In 2 patients, ablation was not performed at epicardial sites close to a coronary artery and phrenic nerve, respectively. In the latter case, pacing at the epicardial exit site stimulated the left phrenic nerve. Although an effort was made to separate the phrenic nerve by distending a 30‐mm vascular balloon inflated in the pericardial space adjacent to the ablation catheter, phrenic capture was still present underneath the balloon. Radiofrequency ablation at adjacent endocardial sites where there was no phrenic nerve stimulation failed to influence the inducible VT. In 37 patients, epicardial ablation was not performed after failed endocardial ablation. In 9 patients, a septal origin was suspected based on the following findings: entrainment showed the closest identifiable ablation site to be part of inner or outer VT loops in 5 patients, earliest endocardial activation was seen on the septum in 7 patients, and VT could be terminated by ablation on the septum but remained inducible afterward in 7 patients (Figure 2). One patient had a septal para‐His VT origin that was not ablated to avoid the risk of AV block. In 27 (52%) patients, the VT circuit may have been epicardial or intramural. Epicardial access was not attempted in favor of further drug trials as per patient or physician preference in 10 patients.

**Figure 3.**
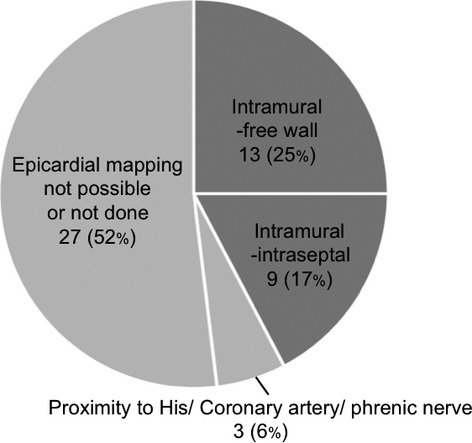
Overall attributed causes of ablation failure are shown.

Overall attributed causes of failure are shown in Figure 3: intramural free wall origin in 13, intramural intraseptal origin in 9, possible intramural or epicardial without epicardial mapping in 27, and protecting structure (His bundle, coronary artery, phrenic nerve) in 3 patients.

Of the 203 patients who had a history of prior cardiac surgery (coronary artery bypass graft in 124, other cardiac surgery in 40, and both in 39), 30 patients were suspected to have an epicardial VT circuit. Of those, percutaneous epicardial access was attained successfully in 10 patients, a surgical subxiphoid pericardial window was used in 3 patients, and percutaneous epicardial access failed in 2 patients and was not attempted in 15 patients.

### Outcomes

Patients with ablation success were more likely to have antiarrhythmic drug doses reduced or discontinued compared with patients after acute ablation failure (42% versus 23%, *P*=0.01) (Table 3). β‐Blockers were usually continued and were not significantly different between those with and without ablation failure (*P*=0.64).

**Table 3.** Drug Treatment Before Ablation and at Follow‐up

	Ablation Failure (n=52)		Ablation Success (n=383)		*P* Value
	Before Ablation	Follow‐Up	Before Ablation	Follow‐Up	
AAD	39 (75)	39 (75)	288 (75)	232 (61)	…
No. of AADs	0.9±0.7	1.0±0.7	1.0±0.7	0.7±0.6	…
Amiodarone	26 (50)	24 (46)	179 (47)	137 (36)	…
Sotalol	7 (14)	8 (15)	78 (20)	70 (18)	…
Dose reduction or discontinuation	12 (23)		162 (42)		0.01
β‐Blocker	35 (67)	37 (71)	280 (73)	294 (77)	…
Metoprolol	16 (31)	24 (46)	135 (35)	152 (40)	…
Carvedilol	16 (31)	9 (17)	117 (31)	101 (26)	…
Dose reduction or discontinuation	11 (21)		64 (17)		0.64

Data are presented as mean±SD or n (%). AAD indicates antiarrhythmic drug.

**Figure 4.**
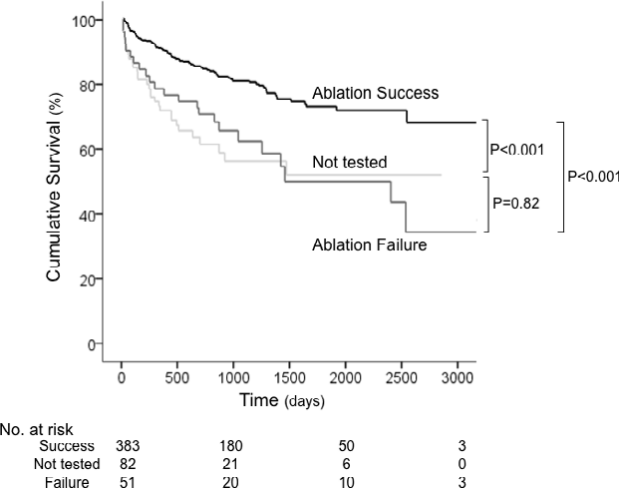
Kaplan–Meier curves showing all cause mortality in the patients with ablation success, those with ablation failure, and those who did not have a postablation attempt to reinduce VT. All 518 patients were included in this survival analysis. In pairwise comparison, Bonferroni adjusted *P*<0.05/3 was considered significant. VT indicates ventricular tachycardia.

**Figure 5.**
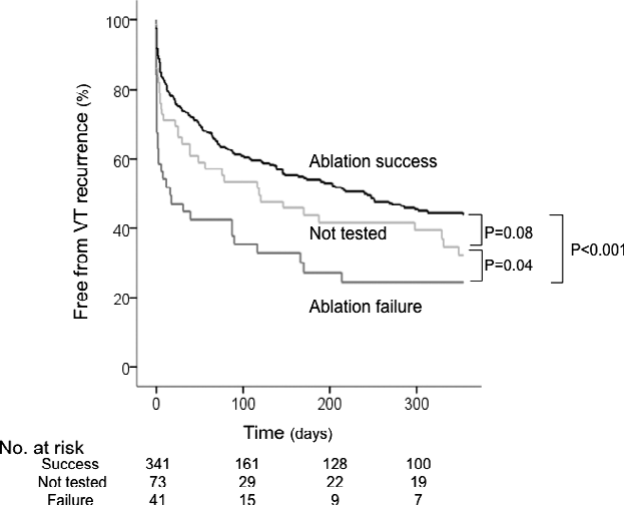
Kaplan–Meier curves showing VT recurrence within 1 year after catheter ablation in the patients with ablation success, those with ablation failure and those who did not have a post ablation attempt to re‐induce VT. All 518 patients were included in this survival analysis. In pairwise comparison, Bonferroni adjusted *P*<0.05/3 was considered significant. VT indicates ventricular tachycardia.

**Figure 6.**
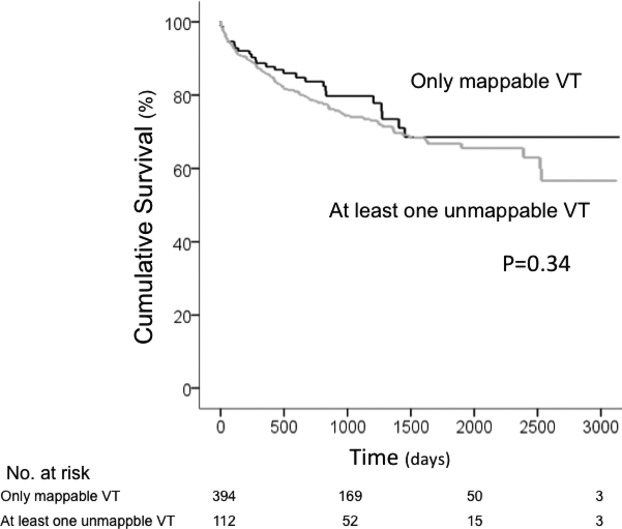
Kaplan–Meier curves showing all cause mortality in the patients with mappable VT only and those with at least 1 unmappable VT. All 518 patients were included in this survival analysis. *P* value is from the log‐rank test. VT indicates ventricular tachycardia.

**Figure 7.**
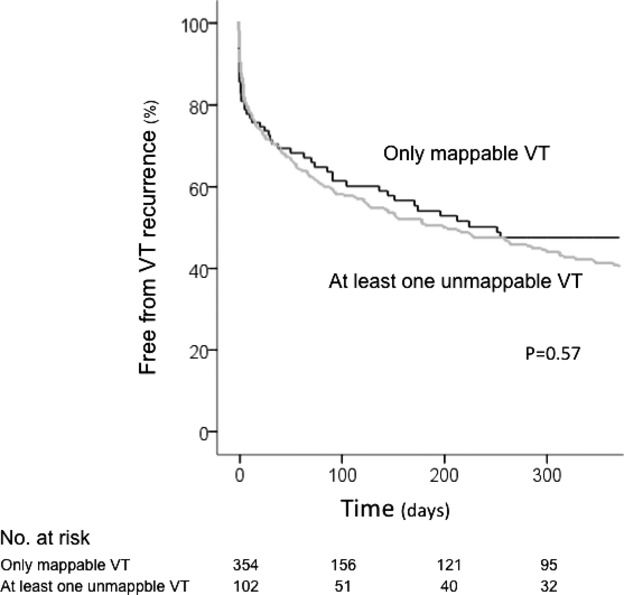
Kaplan–Meier curves showing VT recurrence within 1 year after catheter ablation in the patients with mappable VT only and those with at least 1 unmappable VT. All 518 patients were included in this survival analysis. *P* value is from the log‐rank test. VT indicates ventricular tachycardia.

After ablation, VT recurred in hospital in 22 (42%) of patients with acute failure of ablation. Overall, the 30‐day mortality was 3.2% (17/518) and was due to uncontrollable VT/ventricular fibrillation (VF) with progressive hypotension or cardiac arrest in 11 patients, sepsis after cardiac surgery in 2 patients, and multiorgan failure in 4 patients (Table 4). The 30‐day mortality rate of 8% was due to the patients (4/52) who had with failed ablation as a result of uncontrollable VT/VF. All 518 patients were included in survival analyses (Figures 4 through 7). All‐cause mortality (*P*<0.001, Figure 4) and VT recurrence within 1 year (*P*<0.001, Figure 5) were greater in the patients with ablation failure than in those with ablation success. In the patients who did not have a postablation attempt to reinduce VT, all‐cause mortality (*P*<0.001, Figure 4) and 1‐year recurrence (*P*=0.08, Figure 5) rates were greater than in those with ablation success, although the latter did not reach statistical significance. Neither mortality nor VT recurrence was significantly different between the patients with mappable VT only and those with at least 1 unmappable VT (Figures 6 and 7). In Cox multivariable analysis, ablation failure was an independent predictor of mortality (hazard ratio 1.945, 95% CI 1.217 to 3.108, *P*=0.005, Table 5) and VT recurrence within 1 year (hazard ratio 1.731, 95% CI 1.139 to 2.631, *P*=0.01, Table 6).

**Table 4.** The 30‐Day Mortality

	Acute Success (n=383)	Acute Failure (n=52)	Not Tested (n=83)
30‐d mortality	6 (1.6%)	4 (8%)	7(9%)
Uncontrollable VT/VF	3	4	4
Sepsis after cardiac surgery	2	0	0
Multiorgan failure	1	0	3

Data are presented as n (%). VT indicates ventricular tachycardia; VF, ventricular fibrillation.

**Table 5.** Multivariable Analysis for Mortality

	*P* Value	Hazard Ratio	95% CI
Age	0.01	1.021	1.005 to 1.038
LVEF	<0.001	0.966	0.948 to 0.983
Ischemic cardiomyopathy	0.411	0.823	0.517 to 1.310
NYHA class III or IV	<0.001	2.107	1.421 to 3.123
Renal insufficiency	0.001	2.037	1.339 to 3.098
VT storm	0.989	1.003	0.669 to 1.503
No. of failed AADs	0.625	1.046	0.874 to 1.252
No. of prior failed ablations	0.377	0.899	0.709 to 1.139
Ablation failure	0.005	1.945	1.217 to 3.108
Not tested	0.209	1.492	0.799 to 2.788

LVEF indicates left ventricular ejection fraction; NYHA, New York Heart Association; VT, ventricular tachycardia; AAD, antiarrhythmic drug.

**Table 6.** Multivariable Analysis for VT Recurrence Within 1 Year

	*P* Value	Hazard Ratio	95% CI
Age	0.994	1.000	0.987 to 1.013
LVEF	0.005	0.985	0.974 to 0.995
Ischemic cardiomyopathy	0.052	0.740	0.547 to 1.002
NYHA class III or IV	0.035	1.424	1.026 to 1.978
Renal insufficiency	0.907	1.019	0.740 to 1.404
VT storm	0.324	1.179	0.850 to 1.637
No. of failed AADs	0.018	1.151	1.025 to 1.293
No. of prior VT ablations	0.613	0.952	0.786 to 1.152
Ablation failure	0.010	1.731	1.139 to 2.631
Not tested	0.394	1.232	0.762 to 1.992

VT indicates ventricular tachycardia; LVEF, left ventricular ejection fraction; NYHA, New York Heart Association; AAD, antiarrhythmic drug.

### Repeat Ablation Procedures

Repeat ablation procedures were performed in 155 (30%) patients (Table 7). Success rates in repeat procedure among the groups were similar (*P*=0.90). Of 52 patients with acute ablation failure, repeat ablation procedures were performed in 24 patients; additional advanced ablation procedures were performed using epicardial ablation via subxiphoid surgical window in 6, transcoronary ethanol ablation in 5, and surgical ablation via a thoracotomy in 2. The epicardial ablation and advanced procedures were more frequently performed in the patients with prior ablation failure. Of the 13 patients with additional advanced ablation procedures, clinical VT was abolished in 12 (92%). One year after an advanced ablation procedure, freedom from VT was achieved in 69% (9/13) of the patients.

**Table 7.** Repeat Ablation Procedures

Initial Ablation Short‐term Result	Success (n=383)	Failure (n=52)	Not Tested (n=83)	*P* Value
Repeat ablation procedure	101 (27)	24 (46)	30 (31)	
Epicardial ablation	17 (17)	11 (46)	5 (6)	0.006
Advanced procedure	12 (12)	13 (54)	3 (10)	<0.001
Repeat ablation results				
Success	78 (77)	18 (75)	22 (73)	0.90
Failure	7 (7)	4 (17)	2 (7)	
Nontested	16 (16)	2 (8)	6 (20)	

Data are presented as mean±SD or n (%). Additional advanced procedures included epicardial ablation via subxiphoid surgical window, transcoronary ethanol ablation, and surgical ablation via a thoracotomy.

## Discussion

There are several potential reasons for failure of VT ablation. Ablation can fail if VT is not inducible for mapping and the substrate cannot be defined to allow ablation to be targeted. In these patients, however, it is not possible to clarify anatomic obstacles to ablation, and with substrate‐guided approaches, the acute outcome of the procedure is often uncertain. Therefore, we evaluated only those patients in whom a clinical or presumptive clinical VT was inducible and further characterized the reasons for ablation failure in those with persistently inducible VT despite ablation. To our knowledge, this is the first study to focus on the causes of acute failure of VT ablation. Procedural failure occurred in 10% of patients and was associated with adverse outcomes.

Epicardial or intramural VT origins were the major causes for endocardial ablation failure. Although epicardial approaches are extremely useful for many patients, obstacles remain; failure occurs when the substrate is intramural or protected by adjacent structures. Although the risk of coronary occlusion from ablation procedures is low, occurring in 0.09% of >4000 patients in our previously reported series,^17^ ablation in the coronary sinus and its branches or in the epicardium poses a potential risk of coronary injury. With epicardial ablation, the left phrenic nerve, which descends anterolaterally over the area of the left ventricle to insert in the diaphragm behind the cardiac apex, is also at risk.^18^ Anatomic proximity to the nerve can be detected by pacing with high stimulus strength.^18^ The phrenic nerve can usually be protected by injecting saline or air injection into the pericardium or placement of a balloon catheter between the ablation site and the nerve, although we were not able to achieve this in 1 patient in this series.^19–20^ Percutaneous epicardial access cannot be achieved in all patients, particularly those with prior cardiac surgery.^21^

Intramural substrate was also an important cause of failure. It should be recognized, however, that the presence of intramural VT substrate and origin was largely inferred from the absence of endocardial and epicardial targets, with the closest sites identified suggesting an intramural location. In those patients who did not have epicardial mapping, we cannot distinguish an epicardial source from an intramural source.

In addition to conventional catheter ablation, other advanced approaches, such as epicardial ablation via subxiphoid epicardial window,^14^ transcoronary ethanol ablation,^22–23^ and surgical ablation,^15^ could be considered to address these anatomic obstacles. Irrigated needle‐tipped electrode catheters are also under investigation.^24–25^ Favorable success rates in repeat advanced procedures including epicardial approaches further supported our thesis that anatomic limitation is a major cause of initial failure.

Many studies have described outcomes related to acute ablation results.^3,26–27^ Cardiac death was independently predicted by acute ablation failure in patients with electrical storm.^5^ In several studies, the absence of inducible VT did not reliably predict freedom from recurrent VT during follow‐up.^6,28^ Healing of ablation lesions, changing antiarrhythmic medications after ablation, and variable reporting of clinical and other inducible VTs are likely factors that make the relation of acute outcome to chronic outcome difficult to assess. In the present study, we attempted to focus on a relatively clear ablation failure end point of an inducible clinical or presumptive clinical VT. Ablation failure by this definition predicted mortality and VT recurrence within 1 year. Early mortality after ablation is often due to incessant VT/VF, which might reflect ablation failure to control VT, although a proarrhythmic effect cannot be excluded in some patients.

### Limitations

Most VT recurrences were documented by implantable cardioverter‐defibrillator ECGs rather than 12‐lead ECGs, limiting ability to assess whether recurrent VT was the same as the previous clinical VT, compared with a different VT. Although freedom from any VT is a convenient objective endpoint, a reduction in the burden of VT is also clinically important. Assessing VT burden in our referral population is, however, difficult. As noted earlier, failure due to intramural locations is largely inferred from mapping data, but it can be argued that potentially successful endocardial or epicardial sites were simply missed during mapping in some cases. There are likely important anatomic differences between different forms of heart disease that may be identified in larger cohorts.

## Conclusions

Anatomic constraints and perceived inability to protect adjacent structures still limit catheter ablation procedures for VT. Persistent inducibility of clinical or presumptive clinical VT after ablation is associated with worse clinical outcome.
